# *Lactococcus lactis* carrying the pValac eukaryotic expression vector coding for IL-4 reduces chemically-induced intestinal inflammation by increasing the levels of IL-10-producing regulatory cells

**DOI:** 10.1186/s12934-016-0548-x

**Published:** 2016-08-30

**Authors:** Bianca Mendes Souza, Tatiane Melo Preisser, Vanessa Bastos Pereira, Meritxell Zurita-Turk, Camila Prósperi de Castro, Vanessa Pecini da Cunha, Rafael Pires de Oliveira, Ana Cristina Gomes-Santos, Ana Maria Caetano de Faria, Denise Carmona Cara Machado, Jean-Marc Chatel, Vasco Ariston de Carvalho Azevedo, Philippe Langella, Anderson Miyoshi

**Affiliations:** 1 Laboratório de Tecnologia Genética, Departamento de Biologia Geral, Instituto de Ciências Biológicas, Universidade Federal de Minas Gerais, Belo Horizonte, Minas Gerais Brazil; 2 Departamento de Ciências Biológicas, Instituto Federal do Paraná, Foz do Iguaçu, Paraná Brazil; 3 Laboratório de Imunobiologia, Departamento de Bioquímica e Imunologia, Instituto de Ciências Biológicas, Universidade Federal de Minas Gerais, Belo Horizonte, Brazil; 4 Laboratório de Alergia e Inflamação, Departamento de Morfologia, Instituto de Ciências Biológicas, Universidade Federal de Minas Gerais, Belo Horizonte, Minas Gerais Brazil; 5 INRA, UMR1319 Micalis, Jouy-en-Josas, France; 6 Laboratório de Genética Celular e Molecular Departamento de Biologia Geral, Instituto de Ciências Biológicas, Universidade Federal de Minas Gerais, Belo Horizonte, Minas Gerais Brazil

**Keywords:** *Lactococcus lactis*, Crohn’s disease, Interleukin 4, Interleukin 10, Regulatory cells

## Abstract

**Background:**

Inflammatory bowel diseases are characterized by chronic intestinal inflammation that leads to severe destruction of the intestinal mucosa. Therefore, the understanding of their aetiology as well as the development of new medicines is an important step for the treatment of such diseases. Consequently, the development of *Lactococcus lactis* strains capable of delivering a eukaryotic expression vector encoding the interleukin 4 (IL-4) of *Mus musculus* would represent a new strategy for the elaboration of a more effective alternative therapy against Crohn’s disease.

**Results:**

The murine *IL*-*4* ORF was cloned into the eukaryotic expression vector pValac::dts. The resulting plasmid—pValac::dts::*IL*-*4*—was transfected into CHO cells so that its functionality could be evaluated in vitro. With fluorescent confocal microscopy, flow cytometry and ELISA, it was observed that pValac::dts::*IL*-*4*-transfected cells produced IL-4, while non-transfected cells and cells transfected with the empty vector did not. Then, pValac::dts::*IL*-*4* was inserted into *L. lactis* MG1363 FnBPA^+^ in order to evaluate the therapeutic potential of the recombinant strain against TNBS-induced colitis. Intragastric administration of *L. lactis* MG1363 FnBPA^+^ (pValac::dts::*IL*-*4*) was able to decrease the severity of colitis, with animals showing decreased levels of IL-12, IL-6 and MPO activity; and increased levels of IL-4 and IL-10. Finally, LP-isolated cells from mice administered TNBS were immunophenotyped so that the main IL-4 and IL-10 producers were identified. Mice administered the recombinant strain presented significantly higher percentages of F4/80^+^MHCII^+^Ly6C^−^IL-4^+^, F4/80^+^MHCII^+^Ly6C^−^IL-10^+^, F4/80^+^MHCII^+^Ly6C^−^CD206^+^CD124^+^IL-10^+^ and CD4^+^Foxp3^+^IL10^+^ cells compared to the other groups.

**Conclusions:**

This study shows that *L. lactis* MG1363 FnBPA^+^ (pValac::dts::*IL*-*4*) is a good candidate to maintain the anti-inflammatory and proinflammatory balance in the gastrointestinal tract, increasing the levels of IL-10-secreting regulatory cells and, thus, demonstrating the effectiveness of this novel DNA delivery-based strategy.

## Background

Inflammatory bowel diseases (IBDs) are multifactorial autoimmune disorders of the gastrointestinal (GI) tract, which share many clinical and pathological features but differ in their histological aspects and cytokine profiles [1]. These diseases have a higher incidence and prevalence in Western countries than in Eastern countries, with Western cases having increased rapidly in the last 2–4 decades [2–5]. Moreover, the prevalence of IBDs has increased among children and adolescents [6].

Ulcerative colitis (UC) and Crohn’s disease (CD) are the most important forms of IBDs [7]. Although classified as an intestinal disease, CD can compromise any part of the GI tract, affecting all layers of the intestinal wall, the lymphatic vessels and the mesentery. The most evident histological alterations caused by this disease occur in the submucosal layer, with the formation of caseificant granulomas, inflammatory infiltrate, and exuberant thickening, with a tendency to extend into the peritoneum. This, in turn, favours the formation of fistulas, which is one of the most frequent complications of CD [8]. Additionally, this IBD presents extraintestinal manifestations and sequelae related to the malabsorption of water and nutrients [9, 10].

Since their first modern description 75–100 years ago, these disorders have been enigmatic because their aetiology remains unknown [11]. It is known, however, that these diseases involve the complex interaction of factors associated with the environment, particularities of the intestinal microbiota, and the genotypic, immunological and psychological status of the individual [3, 8]. Thus, the difficulty in establishing the causal agent has compromised the treatment of these disorders, leading to clinical and/or surgical symptomatic treatments [8, 11]. Nevertheless, these treatment proposals have only increased the possibility of improving quality of life, but have not increased the possibility of cure [8].

Discovered in the 1980s, interleukin 4 (IL-4) is a pleiotropic cytokine produced by activated T cells, mast cells, basophils and eosinophils [12, 13]. IL-4 has the ability to exert functions such as the induction of mastocytosis, eosinophilia, goblet cells hyperplasia (increase in mucus production), and immunoglobulin E (IgE) synthesis by B cells [12, 14, 15]. This cytokine also induces the activation of M2 macrophages (mϕ), the functions of which include tissue repair, elimination of parasites, and regulation of inflammation [13, 16]. Additionally, IL-4 participates in the proliferation and differentiation of B and T cells [12, 15, 17], the latter of which is one of its most important functions because it is related to the determination of alternative destinies of T helper (Th) cells, thus promoting their differentiation into Th2 cells and inhibiting their differentiation into Th1 and Th17 cells [13]. This outcome, in turn, has a markedly inhibitory effect on cytokine expression and release by Th1 and Th17 cells, directing immune responses to the Th2 pattern and suppressing the immune responses of the Th1 and Th17 patterns [18, 19].

Therefore, these IL-4 features, together with the development of CD being associated with a breach in oral tolerance, which results in altered regulation of the immune response against commensal microorganisms of the GI tract and generates an imbalance among Th1 (increase), Th2 (decrease) and Th17 (increase) cell populations in the activated state [18, 20], have led to the belief that this cytokine would be a good candidate for the development of new treatments for this IBD.

This hypothesis is even more promising given the results obtained by Hogaboam et al. [18], who tested a therapeutic DNA vaccine composed of a recombinant human type 5 adenovirus (Ad5) carrying the open reading frame (ORF) that encodes murine IL-4. It was suggested that IL-4 had therapeutic effects against acute inflammation—caused by rectal introduction of 2,4,6-trinitrobenzenesulfonic acid (TNBS) dissolved in 50 % ethanol—in the colons of rats when administered, by gene transfer, in two doses. Furthermore, in this study, the therapeutic potential of IL-4 in TNBS-induced colitis was associated with inhibition of the induction of nitric oxide (NO) expression and reduction of its synthesis [18].

Additionally, Xiong et al. [21]. tested a therapeutic DNA vaccine in which a commercial eukaryotic expression vector carrying the ORF that encodes murine IL-4 was administered using a liposomal transfection reagent. It was also suggested that IL-4 had therapeutic effects against acute inflammation caused by rectal introduction of TNBS dissolved in 50 % ethanol in the colons of mice when administered in a single dose [21].

In this context, a new invasive strain of *Lactococcus lactis* that expresses the fibronectin-binding protein A (FnBPA) of *Staphylococcus aureus* was developed. FnBPA is a mediator of the adhesion of *S. aureus* to host tissue and its subsequent entry into non-phagocytic cells. Thus, *L. lactis* MG1363 FnBPA^+^, transformed with a plasmid containing the green fluorescent protein (GFP) coding sequence, was able to enter Caco-2 human epithelial cells more efficiently than the non-invasive strain [22]. Additionally, Caco-2 cells incubated with *L. lactis* MG1363 FnBPA^+^, transformed with a plasmid containing the bovine β-lactoglobulin (BLG) coding sequence, produced 30 times more BLG than cells incubated with the non-invasive strain [23]. Finally, epithelial cells from the small and large intestines of BALB/c mice administered *L. lactis* MG1363 FnBPA^+^ that was transformed with a plasmid containing the GFP coding sequence were able to express GFP in vivo [23, 24].

Furthermore, to improve plasmid DNA delivery strategies, a plasmid called pValac was constructed. This vector was created by fusing (i) the cytomegalovirus promoter (pCMV), which allows for expression of the ORF of interest in eukaryotic cells; (ii) a multiple cloning site (MCS); (iii) the polyadenylation signal sequence of the bovine growth hormone (BGH polyA) to stabilize the messenger RNA (mRNA) transcript; (iv) the origins of replication that allowed for plasmid propagation in both *Escherichia coli* and *L. lactis*; and (v) a chloramphenicol resistance gene for the selection of recombinant strains [25].

Nonetheless, low levels of gene expression have been limiting the potential of DNA vaccines, making the reach of the cell nucleus of the vaccinated individual essential for the ORF of interest to interact with the transcriptional machinery and be expressed. This outcome, in turn, is hampered by many existing cellular barriers. Thus, a new version of the pValac plasmid (pValac::dts) was constructed, into which the simian virus 40 (SV40) DNA nuclear targeting sequence (DTS) was inserted, with the aim of increasing nuclear import levels and consequently, of increasing the expression of the ORF of interest that is present in the plasmid DNA (unpublished observations) (for more details on SV40 DTS, see references [26, 27]).

Therefore, the use of the invasive strain *L. lactis* MG1363 FnBPA^+^ to deliver the pValac::dts plasmid carrying an ORF of interest, such as IL-4, could be a new strategy for the prevention and treatment of several diseases, such as CD.

## Methods

### Bacterial strains and growth conditions

The strains and plasmids that were used are listed in Table 1. *Escherichia coli* TOP10 and *E. coli* TG1 were grown in Luria-Bertani (LB) medium (Accumedia)—with or without ampicillin (100 μg/mL) (Sigma Aldrich), kanamycin (50 μg/mL) (Sigma Aldrich) and chloramphenicol (10 μg/mL) (Sigma Aldrich)—at 37 °C. *Lactococcus lactis* MG1363 and *L. lactis* MG1363 FnBPA^+^ were grown in M17 medium (Fluka Analytical) supplemented with 0.5 % glucose (GM17)—with or without chloramphenicol (5 μg/mL) (Sigma Aldrich) and erythromycin (5 μg/mL) (Sigma Aldrich)—at 30 °C.

**Table 1:** Bacterial strains and plasmids used in this work

	Characteristics	Source
*Strain*		
*Escherichia coli* TOP10	*E. coli* K-12-derived strain; F- mrcA Δ(mrr-hsdRMS-mcrBC) φ80lacZΔM15 ΔlacX74 nupG recA1 araD139 Δ(ara-leu)7697 galE15 galK16 rpsL(Str^R^) endA 1 λ^−^	Life Technologies; Carlsbad, CA/USA
*Escherichia coli* TG1	*E. coli* K-12-derived strain; F’ [*traD36 proAB* ^+^ *lacI* ^q^ *lacZ*Δ*M15*] *supE thi*-*1* Δ(*lac*-*proAB*) Δ(*mcrB*-*hsdSM*)*5* (r_K_^−^ m_K_^−^)	Lucigen; Middleton, MI/USA
*Lactococcus lactis* MG1363 (pValac::dts)	*L. lactis* MG1363 strain carrying the pValac::dts plasmid	This work
*Lactococcus lactis* MG1363 (pValac::dts::*IL*-*4*)	*L. lactis* MG1363 strain carrying the pValac::dts::*IL*-*4* plasmid	This work
*Lactococcus lactis* MG1363 FnBPA^+^	*L. lactis* MG1363 strain expressing *S. aureus* FnBPA	[28]
*Lactococcus lactis* MG1363 FnBPA^+^ (pValac::dts)	*L. lactis* MG1363 FnBPA^+^ strain carrying the pValac::dts plasmid	This work
*Lactococcus lactis* MG1363 FnBPA^+^ (pValac::dts::*IL*-*4*)	*L. lactis* MG1363 FnBPA^+^ strain carrying the pValac::dts::*IL*-*4* plasmid	This work
*Plasmid*		
pUC57::*IL*-*4*	*E. coli* cloning vector (Ap^R^; pMB1 ori; *lac*Z) containing the *Il*-*4* ORF	GenScript; Piscataway, NJ/USA
pCR™-Blunt	*E. coli* cloning vector (Kan^R^; Zeo^R^; pUC ori; P_lac_; *lac*Zα; *ccd*B)	Life Technologies; Carlsbad, CA/USA
pCR™-Blunt::*IL*-*4*	pCR™-Blunt containing the *IL*-*4* ORF	This work
pValac::dts	Eukaryotic expression vector (pCMV; Cm^R^; repA; repC) containing the SV40 DTS	This work
pValac::dts::*IL*-*4*	pValac::dts containing the *IL*-*4* ORF	This work

*Ap* ^*R*^ ampicillin resistance gene, *pMB1 ori* pMB1 origin of replication, *IL-4* interleukin 4, *ORF* open reading frame, *Kan* ^*R*^ kanamycin resistance gene, *Zeo* ^*R*^ zeocin resistance gene, *pUC ori* pUC origin of replication, *P*_*lac*_ lac promoter, *pCMV* cytomegalovirus promoter, *Cm* ^*R*^ chloramphenicol resistance gene, *repA* repA origin of replication, *repC* repC origin of replication, *SV40* Simian virus 40, *DTS* DNA nuclear targeting sequence, *gfp* green fluorescent protein

### pValac::dts::*IL*-*4* construction

The *IL*-*4* ORF of *Mus musculus* was amplified using the synthetic plasmid pUC57::*IL*-*4* (GenScript) as a template. A high fidelity DNA polymerase [Platinum *Pfx* DNA Polymerase (Life Technologies)] and specific oligonucleotides for *IL*-*4* were used [IL4F2: 5′-CTAGCTAGCCCACCATGGGACTGAACCCTCAG-3′; IL4R2: 5′-CGGAATTCTCAGGAATAATCCATCTGCA-3′ (IDT)], with the forward primer (IL4F2) having an artificial restriction site for the *Nhe*I enzyme and the Kozak sequence and the reverse primer (IL4R2) having an artificial restriction site for the *Eco*RI enzyme.

The amplified *IL*-*4* ORF was cloned into the cloning vector pCR-Blunt (Life Technologies), generating the intermediate plasmid pCR-Blunt::*IL*-*4*, which was transformed into *E. coli* TOP10, as described by Reece-Hoyes and Walhout [29]. This vector was digested with both *Nhe*I (Life Technologies) and *Eco*RI (Life Technologies) endonucleases, with the DNA fragment corresponding to the *lL*-*4* ORF purified using the QIAquick Gel Extraction Kit (QIAGEN). The purification product was subcloned into the eukaryotic expression vector pValac::dts, also digested with the aforementioned enzymes and gel purified, resulting in the construction of the therapeutic plasmid pValac::dts::*IL*-*4*, which was transformed into *E. coli* TG1, as also described by Reece-Hoyes and Walhout [29].

The cloning and subcloning events were confirmed by polymerase chain reactions (PCR), enzymatic digestions and sequencing.

### pValac::dts::*IL*-*4* functionality evaluation and verification of IL-4 production and secretion by eukaryotic cells in vitro

#### CHO cells culturing and transfection

Chinese hamster ovary (CHO) cells [Flp-In-CHO cell line (Life Technologies)] were cultured in Nutrient Mixture F-12 Ham medium (Sigma Aldrich) supplemented with 10 % foetal calf serum (FCS) (Life Technologies), 1 % l-glutamine (Sigma Aldrich), 2.5 % 4-(2-hydroxyethyl)piperazine-1-ethanesulfonic acid (HEPES) (Sigma Aldrich) and zeocin (100 ng/mL) (Life Technologies) at 37 °C in 5 % CO_2_.

These cells were transfected with pValac::dts::*IL*-*4* and pValac::dts (negative control) using the Lipofectamine 2000 (Life Technologies) system, as recommended by the manufacturer. Briefly, 90–95 % confluent cells were transfected with 4 μg of plasmidial DNA that was previously complexed with 12 μL of liposomal agent and then they were incubated at 37 °C in 5 % CO_2_ for 48 h.

#### Fluorescence confocal microscopy

CHO cells transfected or not with pValac::dts::*IL*-*4* and pValac::dts (negative control) were fixed with 2 % paraformaldehyde; were permeabilized with 1 % Triton X-100 (Sigma Aldrich); and were stained with an anti-IL-4 primary antibody [IL-4(NYRmIL-4) (Santa Cruz Biotechnology, Inc.)], a secondary antibody conjugated to the Alexa Fluor 488 fluorophore [Alexa Fluor 488 rabbit anti-rat IgG (H + L) conjugate (Life Technologies)], and 4′-6-diamidino-2-phenylindole (DAPI) (Life Technologies), as recommended by the manufacturers.

Images were captured with the Zeiss LSM510 META confocal microscope (Zeiss). The argon laser and filter set 09 were used with an emission wavelength greater than 510 nm to detect the Alexa Fluor 488 fluorophore (Life Technologies), while the epifluorescence and filter set 01 were used to detect DAPI (Life Technologies). All the images were visualized using LSM 5 Image Browser (Zeiss) software.

#### Flow cytometry

CHO cells that were or were not transfected with pValac::dts::*IL*-*4* and pValac::dts (negative control) were fixed and permeabilized with the BD Pharmingen Mouse Foxp3 Buffer Set (BD) and were stained with an anti-IL-4 antibody conjugated to the allophycocyanin (APC) fluorophore (BD Pharmingen APC Rat Anti-Mouse IL-4 [BD]), as recommended by the manufacturers.

Data were acquired with the aid of the BD FACSCalibur flow cytometer (BD) and CellQuest software (BD). The acquired data were analysed using FlowJo software, version 7.6.4 (TreeStar Inc.).

#### ELISA

The 10–30 kDa protein content of the culture supernatants of CHO cells that were or were not transfected with pValac::dts::*IL*-*4* and pValac::dts (negative control) were concentrated with the aid of Amicon Ultra-0.5 10 K (Millipore) and Amicon Ultra-0.5 30 K (Millipore) filter devices, as recommended by the manufacturer. The concentration of secreted IL-4 in the culture supernatants was determined using the BD OptEIA Mouse IL-4 ELISA Set (BD), as recommended by the manufacturer. The sample readings were performed using the Biochrom Asys Expert Plus microplate reader (Biochrom).

### *L. lactis* MG1363 FnBPA^+^ (pValac::dts::*IL*-*4*) strain development

The pValac::dts::*IL*-*4* plasmid was transformed into *L. lactis* MG1363 FnBPA^+^, as described by Langella et al. [30]. To confirm the development of the *L. lactis* MG1363 FnBPA^+^ (pValac::dts::*IL*-*4*) strain by PCR, specific oligonucleotides for *IL*-*4*, the pValac::dts vector [ValF2: 5′- GCTAACTAGAGAACCCACTGCTTACTGG-3′; ValR2: 5′- GCAACTAGAAGGCACAGTCGAGG-3′ (IDT)], and part of the *fnbpA* ORF [FnAF: 5′- CAACACTATTGTGTCCACCG-3′; FnAR: 5′- TCAGCTATTGATATCGATTA-3′ (IDT)] were used. The development of this strain was also confirmed by enzymatic digestions of isolated plasmid DNA.

### Evaluation of the therapeutic potential of *L. lactis* MG1363 FnBPA^+^ (pValac::dts::*IL*-*4*) in a TNBS-induced mouse model of intestinal inflammation

#### Animals

Conventional 6–7-week-old female BALB/c mice were obtained from Centro de Bioterismo (CEBIO) of Universidade Federal de Minas Gerais (UFMG; Belo Horizonte, MG/Brazil). They were kept in collective cages in a controlled environment with a 12-h light–dark cycle and free access to water and food.

#### Induction of intestinal inflammation

The animals, after being subjected to a 6-h fast, were anesthetized by intraperitoneal administration of an anaesthetic/analgesic solution of ketamine (100 mg/kg)/xylazine (10 mg/kg). Intestinal inflammation was induced by intrarectal administration of 100 μL of a mixture consisting of 40 μL of TNBS 5 % (w/v) in H_2_O (Sigma Aldrich), 50 μL of absolute ethanol and 10 μL of phosphate-buffered saline (PBS) 1X in each animal [24]. The procedure was performed once per experiment (day 0).

#### Treatment of intestinal inflammation

To treat TNBS-induced colitis, 100 μL of the corresponding *L. lactis* strain as a suspension—at a concentration of 1 × 10^9^ colony forming units (CFU)/100 μL—in 0.9 % saline was administered by gavage. The intragastric administration of doses was once daily for 4 consecutive days, starting from the day before TNBS administration (days—1 to 2).

Thus, for experimental procedures, animals subjected to TNBS administration were given doses of (i) 0.9 % saline; (ii) *L. lactis* MG1363 (pValac::dts); (iii) *L. lactis* MG1363 (pValac::dts::*IL*-*4*); (iv) *L. lactis* MG1363 FnBPA^+^ (pValac::dts); and (v) *L. lactis* MG1363 FnBPA^+^ (pValac::dts::*IL*-*4*). Additionally, animals that were negative controls for intestinal inflammation were administered doses of 0.9 % saline.

#### Animal health status assessment

Throughout the experiment (days—1 to 3), the animals were examined daily for their behaviour, the state of their fur, body weight, the presence or absence of diarrhoea, and the presence or absence of visible rectal bleeding.

#### Macroscopic and histological evaluation of intestinal inflammation

On the last day of the experiment (day 3), the animals were euthanized, and a macroscopic score of intestinal inflammation was calculated (blind examination), as previously described by Cenac et al. [31]. Characteristics were evaluated such as the presence or absence of adhesions, oedema, stenosis, erythaema, haemorrhage, ulceration, faecal blood, mucus and diarrhoea, with each parameter, except for adhesions and erythaema, were scored one point if observed.

The histological score for intestinal inflammation of haematoxylin and eosin (HE)-stained colon samples was also calculated (blind examination), as previously described by Ameho et al. [32]: histological findings identical to normal mice (grade 0); mild mucosal and/or submucosal inflammatory infiltrate (admixture of neutrophils) and oedema, punctate mucosal erosions often associated with capillary proliferation, muscularis mucosae intact (grade 1); grade 1 changes involving 50 % of the specimen (grade 2); prominent inflammatory infiltrate and oedema (neutrophils usually predominating), frequently with deeper areas of ulceration extending through the muscularis mucosae into the submucosa, rare inflammatory cells invading the muscularis propriae but without muscle necrosis (grade 3); grade 3 changes involving 50 % of the specimen (grade 4); extensive ulceration with coagulative necrosis bordered inferiorly by numerous neutrophils and smaller numbers of mononuclear cells, necrosis extending deeply into the muscularis propriae (grade 5); and grade 5 changes involving 50 % of the specimen (grade 6).

High macroscopic or histological scores indicated increased damage to the intestines.

#### Secretory IgA detection

The concentration of secretory immunoglobulin A (sIgA) in intestinal lavage was determined by a capture ELISA using Goat Anti-Mouse Ig, Human ads-UNLB (Southern Biotech) and Goat Anti-Mouse IgA (α chain specific) Horseradish Peroxidase (HRP) conjugate (Southern Biotech), as recommended by the manufacturer. The sample readings were performed using the Biochrom Asys Expert Plus microplate reader (Biochrom).

#### Cytokine detection

The concentrations of IL-4, interleukin 6 (IL-6), interleukin 10 (IL-10), interleukin 12 (IL-12), interleukin 17A (IL-17A), interferon γ (IFN-γ), transforming growth factor β (TGF-β), and tumour necrosis factor α (TNF-α) in colon homogenates were determined using the BD OptEIA (BD) and R&D DuoSet (R&D Systems) ELISA kits, as recommended by the manufacturers.

However, cytokine concentrations were first normalized to the weight of tissue as follows: colons were homogenized in buffer [23.4 g NaCl; 500 μL Tween 20; 5 g BSA; 34 mg PMSF; 1 mL DMSO; 44.6 mg BC; 372 mg Na_2_EDTA; 40 μL of aprotinin (10 mg/mL) (Sigma-Aldrich); PBS 1X q.s.p. 1 L], with 1 mL of buffer per 100 mg of tissue, and then they were centrifuged at 10,000 rpm for 10 min at 4 °C. The supernatant was subsequently stored at −80 °C until use.

The sample readings were performed using the Biochrom Asys Expert Plus microplate reader (Biochrom).

#### Enzymatic assays

To measure the activity of the enzymes *N*-acetyl-β-d-glucosaminidase (NAG) and myeloperoxidase (MPO), colon samples were homogenized in (i) Buffer I pH 4.7 (0.1 M NaCl; 0.02 M Na_3_PO_4_; 0.015 M Na_2_EDTA); (ii) 0.2 % NaCl and 1.6 % NaCl-5 % glucose; and (iii) buffer II pH 5.4 (0.05 M Na_3_PO_4_; 0.5 % HETAB).

Then, the homogenates were divided in two parts: the first, which was tested for NAG activity, was diluted in citrate–phosphate buffer pH 4.5 (100 mL 0.1 M C_6_H_8_O_7_; 155 mL 0.1 M Na_2_HPO_4_), had the substrate 4-nitrophenyl *N*-acetyl-β-d-glucosaminide (Sigma Aldrich) added and was incubated in the dark at 37 °C for 10 min. The reaction was stopped by adding 0.2 M glycine buffer pH 10.6 (0.8 M glycine; 0.8 M NaCl; 0.8 M NaOH). The sample readings were performed at 405 nm using the Biochrom Asys Expert Plus microplate reader (Biochrom).

The second part of the homogenate, which was tested for MPO activity, was frozen in liquid nitrogen and unfrozen in a room temperature water bath three alternate times. Then, it was diluted in Buffer II pH 5.4, had the substrate 3,3′,5,5′-tetramethylbenzidine (TMB) (Sigma Aldrich) added and was incubated in the dark at 37 °C for 5 min. Subsequently, 0.002 % H_2_O_2_ was added and incubated in the dark at 37 °C for 5 more minutes. The reaction was stopped by adding 1 M H_2_SO_4_, and the sample readings were performed at 450 nm using the Biochrom Asys Expert Plus microplate reader (Biochrom).

### Immunophenotypic characterization of lamina propria IL-4- and IL-10-producing cells

Animals, induction and treatment of intestinal inflammation were the same as in the previous section, except for the experimental groups in which the animals subjected to TNBS administration were given doses of: (i) 0.9 % saline; (ii) *L. lactis* MG1363 FnBPA^+^ (pValac::dts); and (iii) *L. lactis* MG1363 FnBPA^+^ (pValac::dts::*IL*-*4*). Additionally, animals that belonged to the negative control group for intestinal inflammation received doses of 0.9 % saline.

#### Isolation of lamina propria cells

Colons were repeatedly washed in buffers A (HBSS 1X; 5 % FCS; 25 mM HEPES), B (HBSS 1X; 25 mM HEPES; 2 mM EDTA) and C (HBSS 1X; 10 % FCS; 15 mM HEPES; 5 mM EDTA; 0.015 % DTT) for the removal of faeces, mucus and epithelial layer.

Then, small pieces of colon were incubated in Iscove’s Modified Dulbecco’s Medium (IMDM) (Life Technologies) supplemented with 10 % FCS (Life Technologies), 5 % NCTC-109 (Life Technologies), 1 % l-glutamine (Sigma Aldrich), 50 mM 2-mercaptoethanol (Life Technologies), 15 mM HEPES (Sigma Aldrich), gentamicin (50 μg/mL) (Life Technologies), Liberase LT (0085 μg/mL) (Roche Diagnostics) and DNase I (60 μg/mL) (Roche Diagnostics) at 37 °C with shaking for 60 min for tissue digestion.

Finally, digested tissue pieces were pressed through 100 and 40 μm cell strainers (corning) for single-cell suspension obtainment.

#### Flow cytometry

Lamina propria cells (5 × 10^6^ cells) were fixed and permeabilized with the Foxp3/Transcription Factor Staining Buffer Set (Affymetryx eBioscience) and were stained with antibodies for (i) CD45 [Anti-Mouse CD45 APC-eFluor 780 (Affymetryx eBioscience)], F4/80 [PE/Cy7 Anti-Mouse F4/80 Antibody (BioLegend)], MHCII [Biotin Mouse Anti-Mouse I-A[d] (BD)], Ly6C [Anti-Mouse Ly-6C PerCP-Cyanine5.5 (Affymetryx eBioscience)], CD124 [PE Rat Anti-Mouse CD124 (BD)], CD206 [FITC Anti-Mouse CD206 (MMR) Antibody (BioLegend)], and IL-10 [APC Rat Anti-Mouse IL-10 (BD)]; (ii) CD45 [Anti-Mouse CD45 APC-eFluor 780 (Affymetryx eBioscience)], F4/80 [PE/Cy7 Anti-Mouse F4/80 Antibody (BioLegend)], MHCII [Biotin Mouse Anti-Mouse I-A[d] (BD)], Ly6C [Anti-Mouse Ly-6C PerCP-Cyanine5.5 (Affymetryx eBioscience)], CD3 [FITC Rat Anti-Mouse CD3 Molecular Complex (BD)], and IL-4 [PE Rat Anti-Mouse IL-4 (BD)]; and (iii) CD45 [Anti-Mouse CD45 APC-eFluor 780 (Affymetryx eBioscience)], CD4 [PE-Cy7 Rat Anti-Mouse CD4 (BD)], Foxp3 [PE Rat Anti-Mouse Foxp3 (BD)], and IL-10 [APC Rat Anti-Mouse IL-10 (BD)], as recommended by the manufacturers.

Data were acquired with the aid of the BD FACSCanto II flow cytometer (BD) and BD FACSDiva software (BD). The acquired data were analysed using FlowJo software, version 7.6.4 (TreeStar Inc.).

### Statistical analysis

For the evaluation of the therapeutic potential of the invasive *L. lactis* strain carrying the IL-4-coding vector, each experimental group was composed of six animals (four animals were used for the ELISAs and enzymatic assays, and two were used for the histological score of intestinal inflammation), with the results obtained from three experimental replications analysed together. For the immunophenotypic characterization of LP cells, each experimental group consisted of ten animals, with the results obtained from a single experimental replica.

The results were first analysed using the GraphPad QuickCalcs Outlier Calculator online software (http://www.graphpad.com/quickcalcs/Grubbs1.cfm) (GraphPad) to exclude the statistically significant outliers (p < 0.05) that were not due to biological diversity. Then, with the aid of GraphPad Prism software, version 5.0 (GraphPad), an analysis of variance was performed using One-Way ANOVA and Tukey’s post hoc test. Thus, p-values less than 0.05 (p < 0.05) were considered statistically significant.

## Results

### pValac::dts::*IL*-*4* construction

**Fig. 1:**
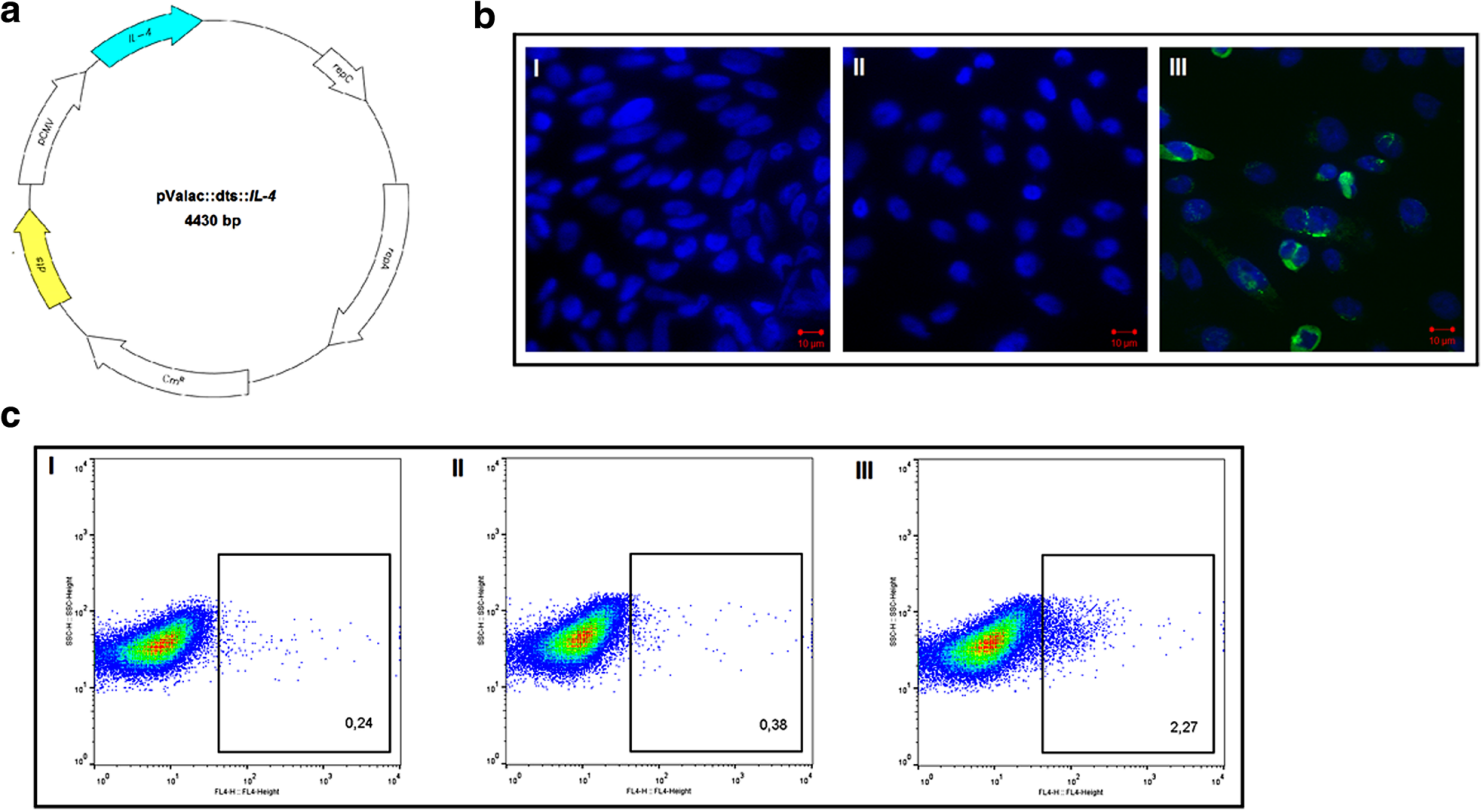
Schematic representation of the eukaryotic expression vector pValac::dts::*IL*-*4* and analysis of its functionality. **a** Schematic representation of pValac::dts::*IL*-*4* showing the DTS (*yellow*), pCMV promoter, *IL*-*4* ORF (*blue*), *E. coli* repC and *L. lactis* repA origins of replication, and the ORF that confers resistance to the antibiotic chloramphenicol (Cm^R^). **b** Photograph of (I) non-transfected CHO cells and cells transfected with (II) pValac::dts and (III) pValac::dts::*IL*-*4* marked with DAPI (Life Technologies; Carlsbad, CA/USA), rat anti-mouse IL-4 (Santa Cruz Biotechnology, Inc.; Dallas, TX/USA), and rabbit anti-rat IgG conjugated with Alexa Fluor 488 (Life Technologies; Carlsbad, CA/USA) antibodies showing the cell nucleus in *blue* and the murine IL-4 in *green*. **c** Flow cytometry acquired events showing (I) non-transfected CHO cells and cells transfected with (II) pValac::dts and (III) pValac::dts::*IL*-*4* marked with APC rat anti-mouse IL-4 antibody (BD; San Jose, CA/USA). The positive events lie within the *rectangles*, together with their percentages. *DTS* DNA nuclear targeting sequence; *pCMV* cytomegalovirus promoter; *IL*-*4* interleukin 4; *ORF* open reading frame; *CHO* chinese hamster ovary cells

The *IL*-*4* ORF was successfully cloned into the eukaryotic expression vector pValac::dts between the pCMV and the BGH polyA site (Fig. 1a), as required for gene expression by host eukaryotic cells. The pValac::dts::*IL*-*4* construction was confirmed by PCR, enzymatic digestions and sequencing (data not shown).

### pValac::dts::*IL*-*4* functionality evaluation and verification of IL-4 production and secretion by mammal cells in vitro

The pValac::dts::*IL*-*4* plasmid was then transfected into CHO cells so that its functionality could be evaluated in vitro. Accordingly, with fluorescence confocal microscopy, at 48 h post-transfection, pValac::dts::*IL*-*4*-transfected cells were able to express murine IL-4, while non-transfected cells and cells transfected with the empty vector were not (Fig. 1b). Additionally, with flow cytometry, it was estimated that 48 h post-transfection, 2.26 % of pValac::dts::*IL*-*4*-transfected cells were able to express murine IL-4 compared to 0.24 % of non-transfected cells and 0.38 % of cells transfected with the empty vector (Fig. 1c). Finally, with ELISA, murine IL-4 was only detected at 48 h post-transfection in the concentrated culture supernatant of pValac::dts::*IL*-*4*-transfected cells (5.25 ± 0.1 ng/mL) compared to the concentrated culture supernatant of non-transfected cells and cells transfected with the empty vector (data not shown).

### *L. lactis* MG1363 FnBPA^+^ (pValac::dts::*IL*-*4*) strain development

The pValac::dts::*IL*-*4* plasmid was successfully transformed into the invasive strain *L. lactis* MG1363 FnBPA^+^, which is a recombinant strain capable of adhering to and invading eukaryotic cells [22]. The *L. lactis* MG1363 FnBPA^+^ (pValac::dts::*IL*-*4*) strain development was confirmed by PCR and enzymatic digestions of isolated plasmid DNA (data not shown).

### Evaluation of the therapeutic potential of *L. lactis* MG1363 FnBPA^+^ (pValac::dts::*IL*-*4*) in the TNBS-induced mouse model of intestinal inflammation

#### Animal health status assessment

Throughout the experiment, the health status of the animals was assessed. Thus, all the animals subjected to TNBS administration—except for those that received doses of *L. lactis* MG1363 FnBPA^+^ (pValac::dts::*IL*-*4*) (FI group)—presented reduced mobility, hunched-back posture, piloerection, diarrhoea and visible rectal bleeding, while the animals that belonged to the negative control and FI groups did not (data not shown).

**Fig. 2:**
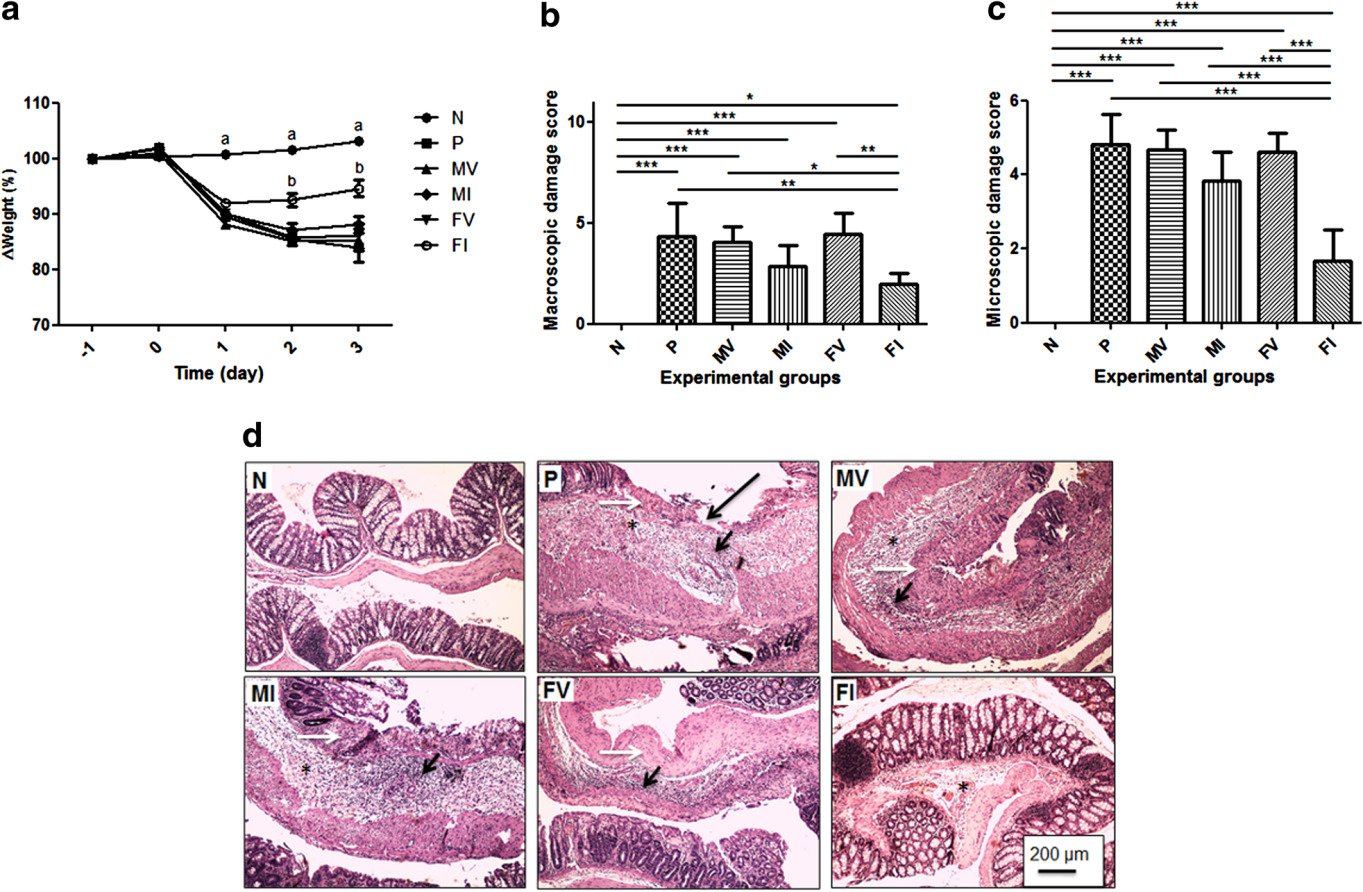
Body weight variations, damage scores, and histological aspects of colonic tissue from mice administered TNBS. **a** Percentage of initial body weight of BALB/c mice subjected or not to TNBS administration as function of time (n = 18). **b** Macroscopic damage score of TNBS-induced colitis in BALB/c mice (n = 18). **c** Microscopic damage score of TNBS-induced colitis in BALB/c mice (n = 6). **d** Histological aspect of colonic tissue from BALC/c mice subjected or not to TNBS administration. *Long black arrow* ulceration; *white arrow* necrosis; *star* edema; and *short black arrow* inflammatory infiltrate. N, negative control; P, positive control; MV, mice that received *L. lactis* MG1363 (pValac::dts) intragastrically; MI, mice that received *L. lactis* MG1363 (pValac::dts::*IL*-*4*) intragastrically; FV, mice that received *L. lactis* MG1363 FnBPA^+^ (pValac::dts) intragastrically; and FI, mice that received *L. lactis* MG1363 FnBPA^+^ (pValac::dts::*IL*-*4*) intragastrically. **a** Experimental group whose percentage of initial body weight is significantly different from the percentage of groups P, MV, MI, FV and FI (p < 0.001). **b** Experimental group whose percentage of initial body weight is significantly different from the percentage of groups N, P, MV, MI and FV (p < 0.001). Data are shown as mean ± SD. *p* value: *p < 0.05; **p < 0.01; ***p < 0.001

All the animals subjected to TNBS administration presented significant weight loss on day 1 compared to the negative control group. However, on days 2 and 3, the FI group presented significant weight gain, while the other groups subjected to TNBS administration continued to lose weight and/or maintained their weights close to those of the previous day (Fig. 2a).

#### Macroscopic and histological evaluation of intestinal inflammation

To assess the severity of lesions caused by intrarectal TNBS administration, macroscopic and microscopic damage scores were calculated. The negative control group presented neither macroscopic (Fig. 2b) nor microscopic intestinal lesions (Fig. 2c, dI). HE staining showed that this group presented an intact mucosal layer with an adequate proportion of goblet cells, thin submucosal and serosal layers and muscular layer thickness compatible with the analysed segment. In addition, signs of degenerative, vascular, inflammatory or pathological cell proliferation processes were not observed (Fig. 2dI).

All the animals subjected to TNBS administration– except for those that belonged to the FI group—presented similar macroscopic (Fig. 2b) and microscopic damage scores (Fig. 2c). HE staining showed that these groups presented a compromised histological architecture; despite the layers being evident, areas of ulceration and necrosis, the absence of goblet cells, and severe erosion of the epithelium were observed in the mucosal layer; and marked oedema with diffuse inflammatory infiltrate was observed in the submucosal layer (Fig. 2dII–V).

The FI group, however, presented significantly reduced macroscopic damage compared to the animals that belonged to the positive control group for intestinal inflammation and the groups that received doses of *L. lactis* carrying the empty vector (Fig. 2b). Additionally, the FI group presented significantly reduced microscopic damage compared to the other groups that were subjected to TNBS administration (Fig. 2c). Thus, HE staining showed that the FI group presented significant improvement of histological patterns: small areas of erosion and small areas with few goblet cells in the mucosal layer, reduced areas of oedema and no inflammatory infiltrate in the submucosal layer (Fig. 2dIV).

#### Assessment of local induced immune responses

To assess whether the administration of *L. lactis* MG1363 FnBPA^+^ (pValac::dts::*IL*-*4*) was able to alter immune responses in the GI tract, levels of anti- and proinflammatory mediators were estimated.

**Fig. 3:**
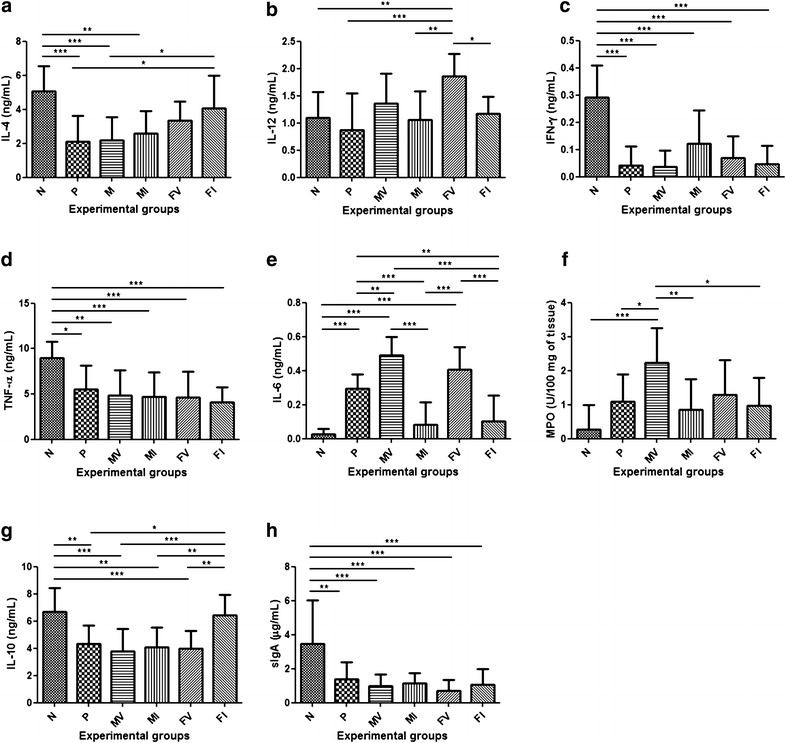
Colonic cytokines, sIgA and MPO activity levels from BALB/c mice that had TNBS administered intrarectally. **a** Interleukin 4 (IL-4) levels. **b** Interleukin 12 (IL-12) levels. **c** Interferon γ (IFN-γ) levels. **d** Tumor necrosis factor α (TNF-α) levels. **e** Interleukin 6 (IL-6) levels. **f** Mieloperoxidase (MPO) activity levels. **g** Interleukin 10 (IL-10) levels. **h** Secretory immunoglobulin A (sIgA) levels. N, negative control; P, positive control; MV, mice that received *L. lactis* MG1363 (pValac::dts) intragastrically; MI, mice that received *L. lactis* MG1363 (pValac::dts::*IL*-*4*) intragastrically; FV, mice that received *L. lactis* MG1363 FnBPA^+^ (pValac::dts) intragastrically; and FI, mice that received *L. lactis* MG1363 FnBPA^+^ (pValac::dts::*IL*-*4*) intragastrically. Data are shown as mean ± SD (n = 12). *p* value: *p < 0.05; **p < 0.01; ***p < 0.001

IL-4 levels are usually reduced both in CD and the TNBS-induced model of colitis [33, 34]. Thus, the animals subjected to TNBS administration—except for those that belonged to the FI group—presented lower levels of this cytokine than the negative control and FI groups (Fig. 3a).

In contrast, the production of IL-12 by lamina propria (LP) antigen-presenting cells (APCs) is usually increased in this model of intestinal inflammation [34]. Interestingly, the animals that received doses of the invasive *L. lactis* strain carrying the empty vector (FV group) presented a significant increase in the levels of IL-12 compared to all the other animals. However, compared to the animals that received doses of *L. lactis* carrying the empty vector, those that received doses of *L. lactis* carrying the IL-4-coding vector presented a decrease in the levels of this proinflammatory cytokine (Fig. 3b).

To observe whether the production of IL-12 by APCs induced IFN-γ production by CD4^+^ T cells, levels of IFN-γ were measured. All the groups presented low levels of IFN-γ, especially those subjected to TNBS administration (Fig. 3c). Similarly, the animals subjected to administration of the haptenization agent presented lower TNF-α levels than the negative control group (Fig. 3d).

IL-6 could also be an inflammatory mediator responsible for causing/maintaining the colitis that was observed in the animals subjected to TNBS administration. Thus, the animals that belonged to the positive control group and those that received doses of *L. lactis* carrying the empty vector presented a significant increase in the levels of IL-6 compared to the animals in the negative control group and those that received doses of *L. lactis* carrying the IL-4-coding vector (Fig. 3e).

Because IL-6 appears to be intimately involved in regulating the transition of neutrophil to monocyte recruitment [35, 36] MPO and NAG enzymatic activities were also measured. Similar to IL-6, the positive control group and the animals that received doses of *L. lactis* carrying the empty vector presented higher MPO activity levels than the negative control group and the animals that received doses of *L. lactis* carrying the IL-4-coding vector (Fig. 3f). However, all the groups presented equivalent NAG activity (data not shown).

In addition, because IL-6 signalling, along with TGF-β, is fundamental for Th17 cell differentiation [37, 38] IL-17A levels were measured. Nevertheless, this cytokine was only detected at baseline levels (15.6 ± 23.3 pg/mL) in the negative control group (data not shown).

Although TGF-β is important for Th17 cell differentiation, T cells that receive signals from this cytokine can differentiate into either Th17 or regulatory T (Treg) cells [39]. Thus, it was possible to observe that all the groups were able to express TGF-β, but none of the groups showed significantly different levels of this cytokine (data not shown).

However, an interesting fact is that IL-10 is required for the maintenance and/or effectiveness of TGF-β-mediated regulatory responses [40, 41]. Thus, similar to IL-4, animals subjected to TNBS administration—except those in the FI group—presented a significant decrease in the levels of IL-10 compared to the negative control and FI groups (Fig. 3g).

Finally, because IgA is essential for the first line of defence against pathogens on mucosal surfaces [42, 43], levels of sIgA were measured. Nevertheless, all the animals subjected to TNBS administration presented lower sIgA levels than the negative control group (Fig. 3h).

### Immunophenotypic characterization of lamina propria IL-4- and IL-10-producing cells

**Fig. 4:**
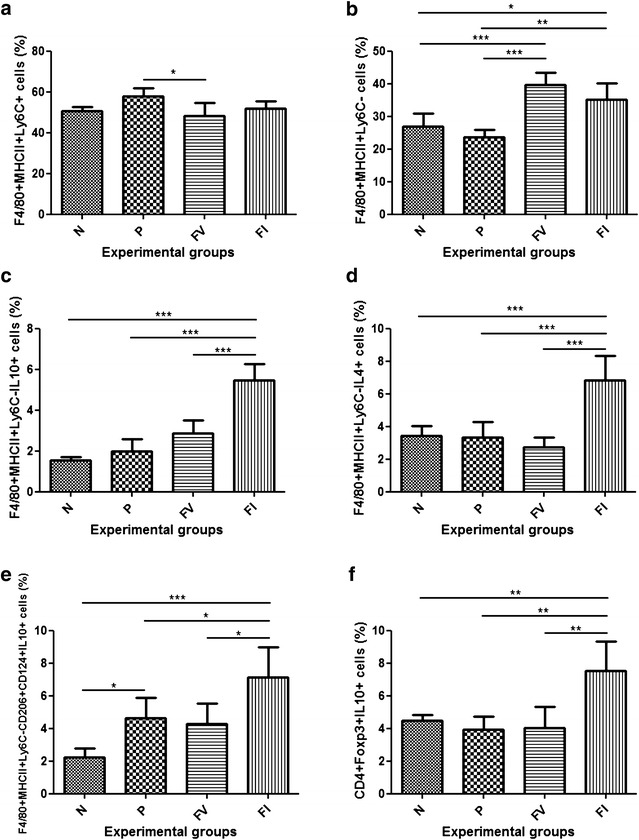
Immunophenotypic characterization of lamina propria IL-4- and IL-10-producing cells. **a** Percentage of F4/80^+^MHCII^+^Ly6C^+^ cells. **b** Percentage of F4/80^+^MHCII^+^Ly6C^−^ cells. **c** Percentage of F4/80^+^MHCII^+^Ly6C^−^IL-4^+^ cells. **d** Percentage of F4/80^+^MHCII^+^Ly6C^−^IL-10^+^ cells. **e** Percentage of F4/80^+^MHCII^+^Ly6C^−^CD206^+^CD124^+^IL-10^+^ cells. **f** Percentage of CD4^+^Foxp3^+^IL-10^+^ cells. N, negative control; P, positive control; FV, mice that received *L. lactis* MG1363 FnBPA^+^ (pValac::dts) intragastrically; and FI, mice that received *L. lactis* MG1363 FnBPA^+^ (pValac::dts::*IL*-*4*) intragastrically. Data are shown as mean ± SD (n = 10). *p* value: *p < 0.05; **p < 0.01; ***p < 0.001

To investigate which cells could produce IL-4 and IL-10 in the colons of mice that received doses of *L. lactis* MG1363 FnBPA^+^ (pValac::dts::*IL*-*4*), LP IL-4- and IL-10-producing cells were immunophenotyped. Thus, the animals that belonged to the FI group did not appear to recruit more F4/80^+^MHCII^+^Ly6C^+^ monocytes than the other groups (Fig. 4a). Nevertheless, the animals that received doses of the invasive *L. lactis* strain—with or without the IL-4-coding vector—presented more F4/80^+^MHCII^+^Ly6C^−^ mϕ (Fig. 4b).

Interestingly, the FI group presented a significantly higher percentage of IL-4- and IL-10-producing mϕ (F4/80^+^MHCII^+^Ly6C^−^IL-4^+^ and F4/80^+^MHCII^+^Ly6C^−^IL-10^+^ cells) than the other groups (Fig. 4c, d). Notably, however, this group also presented a significant increase in the number of F4/80^+^MHCII^+^Ly6C^−^CD206^+^CD124^+^IL-10^+^ mϕ (Fig. 4e).

Furthermore, the animals that belonged to the FI group presented a significantly higher percentage of CD4^+^Foxp3^+^IL-10^+^ cells than the other groups (Fig. 4f).

## Discussion

Over the years, some research groups have attempted to develop alternative treatments for IBDs using therapeutic DNA vaccines for the delivery of the *IL*-*4* ORF to host cells. Hogaboam et al. [18] tested a therapeutic DNA vaccine in which a recombinant Ad5 that carried the *IL*-*4* ORF was intraperitoneally administered to rats. Similarly, Xiong et al. [21] tested a therapeutic DNA vaccine in which a eukaryotic expression vector containing the *IL*-*4* ORF was intraperitoneally administered to mice using the Lipofectamine 2000 (Life Technologies) system. Thus, oral administration of a therapeutic DNA vaccine in which a eukaryotic expression vector containing a DTS and the *IL*-*4* ORF was carried by the model LAB appeared to be a promising strategy because it used a non-invasive delivery route directed to the site of inflammation.

To this end, the pValac::dts::*IL*-*4* plasmid was constructed (Fig. 1a) and its functionality was confirmed by fluorescent confocal microscopy (Fig. 1b), flow cytometry (Fig. 1c) and ELISA. Then, it was transformed into the invasive strain *L. lactis* MG1363 FnBPA^+^ so that the therapeutic potential of this new construction, *L. lactis* MG1363 FnBPA^+^ (pValac::dts::*IL*-*4*), could be evaluated in the acute TNBS-induced mouse model of colitis.

As expected, animals that received doses of *L. lactis* MG1363 FnBPA^+^ (pValac::dts::*IL*-*4*) presented decreased severity of intestinal inflammation with no visible signs of pain, less body weight loss (Fig. 2a), and lower macroscopic and microscopic inflammatory scores (Fig. 2b, c).

Thus, to assess whether the anti-inflammatory potential of this strain was due to changes in the intestinal immune profile, anti- and proinflammatory mediators were analysed. The administration of *L. lactis* MG1363 FnBPA^+^ (pValac::dts::*IL*-*4*) to animals subjected to TNBS-induced colitis led to an increase in IL-4 expression (Fig. 3a), tending towards the restoration of baseline levels of this cytokine and hence contributing to the recovery of homeostasis. This outcome, in turn, indicated that the *IL*-*4* ORF present in the MCS of pValac::dts was transcribed, translated and secreted by the host cells and, somehow, was able to alter that microenvironment.

Because TNBS-induced colitis leads to marked inflammation in a pattern that is similar to that described for CD [34], the levels of the proinflammatory cytokines IL-12, IFN-γ, TNF-α, IL-6 and IL-17A were analysed. Thus, the probable contact of *L. lactis* with APCs appeared to have induced an increase in IL-12 production by these cells (Fig. 3b). However, it could also suggest that animals that received doses of the invasive strain carrying the empty vector were able to produce higher levels of IL-12 because this strain expresses the FnBPA protein—which mediates adhesion of bacteria to tissue and its subsequent entry into host cells—increasing the number of invaded cells and/or the number of CFU that invaded the same cell and being, thereby, capable of stimulating a greater number of APCs to produce IL-12 and/or to induce the same number of APCs to produce higher levels of this cytokine. In addition, as reported by Levings and Schrader [44], IL-4 appears to reduce the production of IL-12 by APCs (monocytes and mϕ) stimulated by bacterial products, with this reduction dependent or not on the STAT6 pathway. This factor, in turn, appears to downregulate the production of proinflammatory cytokines, such as IL-12, by competing with the transcription factor nuclear factor ΚB (NF-ΚB), DNA-binding activity or the expression of proteins that lower the levels of these cytokines [44].

In contrast, the time of sampling did not seem to have coincided with the peak production of IFN-γ and TNF-α (Fig. 3c, d). The low IFN-γ levels produced by animals subjected to TNBS administration could be due to the period of time spent by LP CD4^+^ T cells to express the transcription factor T-box expressed in T cells (T-bet), which appears to be critical to the initiation and perpetuation of colitis mediated by Th1 cells [45] and whose action appears to precede the IL-12/STAT4 pathway. Once stimulated, naïve T cells activate T-bet, which coordinates its own induction, chromatin remodelling of IFN-γ alleles and expression of IL-12Rβ2 receptors [46]. Nevertheless, after being stimulated, T cells require up to 72 h to produce increased amounts of T-bet-coding transcripts and, consequently, of IFN-γ [45, 47], being the levels of this cytokine measured, in the present study, approximately 72 h after TNBS administration. Furthermore, the production of mRNA molecules coding for proinflammatory cytokines, such as TNF-α, peaks between 2 and 3 days after TNBS administration and, with the exception of IL-12, returns to baseline levels within 5 days [48]. Thus, because TNF-α levels were measured, in the present study, 3 days after administration of the haptenization agent, low levels of this cytokine were expected in the colons of animals subjected to TNBS administration.

The increased production of IL-6 by animals that belonged to the positive control group for intestinal inflammation and those that received doses of *L. lactis* carrying the empty vector (Fig. 3e) was consistent with what has been described because high levels of this cytokine are not only correlated to the activity of IBDs and are used as prognostic markers but also play an important functional role in their pathogenesis [38]. One example is the study by Yamamoto et al. [49], in which treatment of murine colitis induced by adoptive transfer of CD4^+^ CD45RB^hi^ T cells with antibodies to IL-6 receptor (IL-6R) reduced T cell expansion and attenuated the expression of molecules that promote interaction between leucocytes and endothelial cells, such as intercellular adhesion molecule 1 (ICAM-1) and vascular cell adhesion molecule 1 (VCAM-1), which are required for cell infiltration. Similarly, Atreya et al. [33] showed that specific inhibition of IL-6 trans-signalling was capable of inducing T cell apoptosis and thereby protected against TNBS-induced colitis and colitis in animals deficient for IL-10 (IL-10^−/−^).

Moreover, IL-6 appears to favour infiltration of neutrophils and initiation of the immune response during acute inflammation, favour infiltration of mononuclear cells—such as mϕ and lymphocytes—and participate in disease pathogenesis during chronic inflammation [35, 36]. Thus, the fact that animals that belonged to the positive control group for intestinal inflammation and the groups that received doses of *L. lactis* carrying the empty vector presented higher levels of MPO activity (Fig. 3f), and that all groups presented similar levels of NAG activity suggested that the colitis that was observed in animals subjected to TNBS administration was still in the acute phase.

Accordingly, one possible explanation for the undetectable levels of IL-17A in animals subjected to TNBS administration is that increased production of this cytokine can only be observed from the transition of the acute to the chronic phase of colitis [34], which did not seem to be the case of this study. However, Jin et al. [50] demonstrated that IL-17 neutralization also contributed to inflammation because it resulted in downregulation of claudin expression and mucin secretion. This outcome, in turn, decreased mucosal barrier function and stimulated T cell activation, because, in this manner, TNBS and bacterial antigens present in the gut lumen had easy access to APCs [50].

The induction of the transcription factor forkhead box P3 (Foxp3) can restrain differentiation of Th17 cells in response to TGF-β in the absence of proinflammatory cytokines by inhibiting the activity of the transcription factor RAR-related orphan receptor γt (RORγt). Conversely, suppression of Foxp3 expression and activity, along with upregulation and stabilization of RORγt expression in the presence of proinflammatory cytokines, might favour the development of Th17 cells [39]. Additionally, Foxp3^+^ cells generated in the presence of TGF-β appear to be inversely related to environmental levels of IL-6 [51]. However, there was no difference in TGF-β levels among groups.

Opposite to TGF-β, but similar to IL-4, animals that received doses of *L. lactis* MG1363 FnBPA^+^ (pValac::dts::*IL*-*4*) were the only ones among those subjected to TNBS administration that showed an increase in IL-10 production sufficient to restore its baseline levels. These results were consistent with what has been described, especially by Kitani et al. [40] and Fuss et al. [41], who demonstrated that IL-10 is important for the expansion of TGF-β-producing regulatory cells and for TGF-β secretion, affecting not the induction phase but the effector phase of these cells. The production of high levels of TGF-β occurs in environments in which production of Th1 cytokines is relatively low, with IL-10 the main cytokine responsible for regulating the production of such proinflammatory cytokines. IL-10 also appears to be required for the responsiveness of other cells to the regulatory effects of TGF-β because activated cells have reduced expression of the TGF-β2R receptor, which can be reversed by IL-10 [41, 52].

Additionally, the immunomodulatory potential of *L. lactis* strains carrying the IL-10-coding vector pValac::*IL*-*10* has been successfully shown in colitis induced by both TNBS [24] and dextran sulphate sodium (DSS) [53]. First, Del Carmen et al. [24] demonstrated that mice administered *L. lactis* MG1363 FnBPA^+^ (pValac::*IL*-*10*) had decreased severity of TNBS-induced intestinal inflammation likely due to increased IL-10 levels and decreased IFN-γ and IL-17 levels, compared to the other groups subjected to administration of the haptenization agent. Then, Zurita-Turk et al. [53] demonstrated that mice administered either the non-invasive strain *L. lactis* MG1363 (pValac::*IL*-*10*) or the invasive strain *L. lactis* MG1363 FnBPA^+^ (pValac::*IL*-*10*) had decreased severity of DSS-induced intestinal inflammation likely due to increased IL-10 levels and decreased IL-6 levels, compared to the positive control for colitis. Thus, direct delivery of high levels of IL-10 also appears to downregulate Th1-and Th17-mediated inflammation.

Furthermore, because sIgA is considered vital for communication between the intestinal microbiota and the immune system [42, 43], the animals subjected to TNBS administration presented lower levels of this immunoglobulin, which suggested that the decrease in sIgA levels could have contributed to the onset of the inflammatory disorders observed in this study. However, it also indicated that sIgA likely did not participate in the immune response to acute TNBS-induced colitis.

Thus, to assess the cells that could be responsible for producing IL-4 and IL-10 in the colons of mice that received doses of *L. lactis* MG1363 FnBPA^+^ (pValac::dts::*IL*-*4*), LP-isolated cells were immunophenotyped. These animals presented the highest percentages of F4/80^+^MHCII^+^Ly6C^−^IL-4^+^ cells (Fig. 4c), indicating that it was likely that resident mϕ—positioned immediately under the epithelial monolayer and ideally located to capture any material that breaches the epithelial barrier [54–56]—were the main cells that interacted with the invasive *L. lactis* strain carrying the IL-4-coding vector. This finding, however, does not exclude the possibility that these bacteria interacted with classically activated mϕ and/or other cell types.

Similarly, animals that received doses of *L. lactis* MG1363 FnBPA^+^ (pValac::dts::*IL*-*4*) presented the highest percentages of F4/80^+^MHCII^+^Ly6C^−^IL-10^+^ cells (Fig. 4d), and although the CX3CR1 marker was not used to distinguish CX3CR1^hi^ (resident mϕ) from CX3CR1^int^ (classically activated mϕ) cells, it is believed that such IL-10-producing cells are resident mϕ, which are constitutive producers of this anti-inflammatory cytokine [54–56]. Supporting this hypothesis is that, similar to what was observed in this study, both CX3CR1^int^ cell and neutrophil compartments appears to contract to homeostatic levels during the resolution of intestinal inflammation [55]. Additionally, the other animals subjected to TNBS administration presented fewer (but not absence of) F4/80^+^MHCII^+^Ly6C^−^IL-10^+^ cells (Fig. 4d) and produced lower levels of IL-10 (Fig. 3g) and higher levels of IL-6 (Fig. 3e). This finding, in turn, agreed with the idea that during intestinal inflammation, the normal balance between CX3CR1^hi^ and CX3CR1^int^ cells is reversed, with infiltrating CX3CR1^int^ cells showing typical proinflammatory characteristics—which includes IL-6 production—and the remaining CX3CR1^hi^ cells retaining their anti-inflammatory characteristics—which includes IL-10 production [55, 56].

Although mϕ are usually classified as M1 (classically activated mϕ) and M2 (alternatively activated mϕ), resident mϕ do not fit readily into this classification because they have characteristics of both types, such as expression of high levels of MHCII and TNF-α (M1) and expression of CD206 and IL-10 (M2) [56]. However, a unique characteristic of M2 mϕ appears to be the expression of the IL-4 receptor CD124 [54]. Therefore, it was interesting to observe that the animals that received doses of the invasive *L. lactis* strain carrying the IL-4-coding vector also showed a higher percentage of F4/80^+^MHCII^+^Ly6C^−^CD206^+^CD124^+^IL-10^+^ cells (Fig. 4e).

Nevertheless, because M2 mϕ are cells with remarkable differences in biochemistry and physiology, they can be further divided into (i) wound-healing mϕ and (ii) regulatory mϕ. The former arise in response to IL-4 and express the antigen resistin-like molecule α (RELMα)—which can promote deposition of extracellular matrix—and produce low levels of IL-10 and IL-12, and the latter arise in response to various stimuli, such as immune complexes, apoptotic cells and IL-10, and produce high levels of IL-10 and low levels of IL-12. Additionally, due to mϕ plasticity, IL-4-primed cells treated with LPS and immune complexes can become hybrid mϕ able to express RELMα and to produce high levels of IL-10 and low levels of IL-12 [57], more closely resembling what was observed in this study.

Moreover, as previously described by Mowat and Bain [54], the IL-10 produced by these mϕ could have promoted further clonal expansion and terminal differentiation of Treg cells in the colons of animals that received doses of *L. lactis* MG1363 FnBPA^+^ (pValac::dts::*IL*-*4*) because they had a higher percentage of CD4^+^Foxp3^+^IL-10^+^ cells than the other groups (Fig. 4f). These cells, in turn, are needed to prevent inflammatory reactions against commensal bacteria and food proteins [54].

**Fig. 5:**
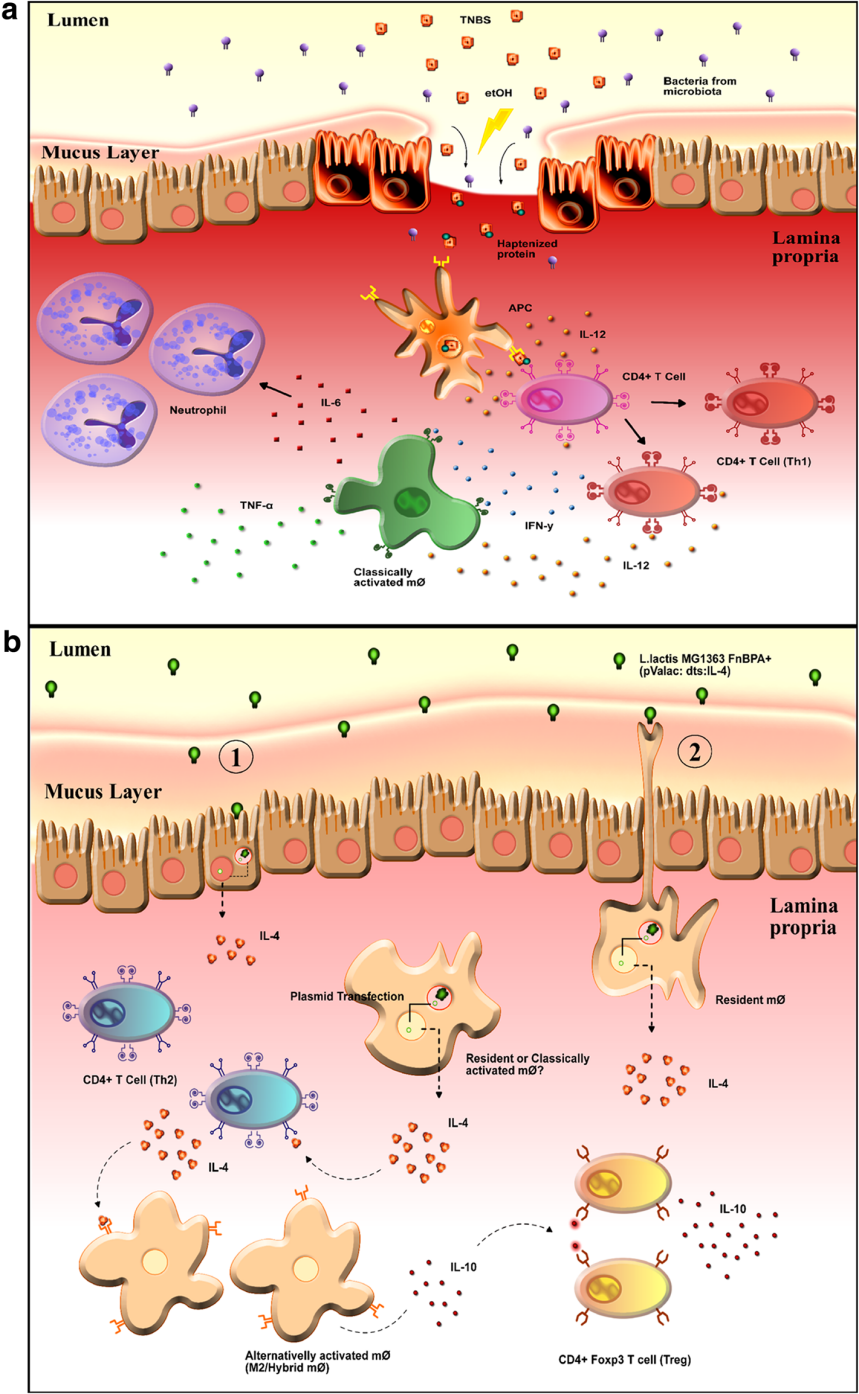
Schematic representation of the proposed mechanisms of action of TNBS and *L. lactis* FnBPA^+^ (pValac::dts::*IL*-*4*). **a** In TNBS-induced colitis, ethanol (etOH) disrupts the mucosal barrier and allows translocation of 2,4,6-trinitrobenzenesulfonic acid (TNBS). The haptenization agent interacts with amine groups of colon and/or microbiota protein, binding trinitrophenyl groups to these proteins indiscriminately. Haptenized proteins are engulfed, processed and presented by antigen presenting cells (APCs) to T cells in the lamina propria. In the absence of appropriate regulatory mechanisms, this leads to excessive secretion of interleukin 12 (IL-12) by classically activated macrophages (mϕ) and induce an exaggerated T helper 1 (Th1) response—production of high levels of interferon γ (IFN-γ)—that leads to the production of other proinflammatory cytokines such as tumor necrosis factor α (TNF-α) and interleukin 6 (IL-6) by mϕ, which are the immediate cause of the intestinal inflammation [28, 52]. **b** Administration of *L. lactis* MG1363 FnBPA^+^ (pValac::dts::*IL*-*4*) possibly leads to its entry into epithelial cells (1) and/or transepithelial dendrites extended by resident mϕ to capture antigens in the lumen (2). This bacterium is then lysed and pValac::dts::*IL*-*4* is released in the cytoplasm and translocated to the nucleus where the expression of the IL-4-coding sequence occurs [53]. Interleukin 4 (IL-4) secreted by transfected cells may contribute to the differentiation of T helper 2 (Th2) cells and consequently, may cooperate with the restoration of the levels of this cytokine which has a markedly inhibitory effect on the expression and release of Th1 cytokines [13]. Combination of IL-4 and bacterial products could then lead to the development of an alternatively activated mϕ population that participates in tissue remodeling and repair and produces high levels of the immunosuppressive cytokine interleukin 10 (IL-10). Through IL-10 production, these M2/hybrid mϕ appear to facilitate secondary expansion and maintenance of IL-10-producing Foxp3^+^ T regulatory (Treg) cells and consequently, regulate TNBS-induced colitis [49, 51, 54, 55]

Therefore, based on what has been described in the literature and the results obtained in this study, it is possible to suggest that ethanol first disrupts the mucosal barrier of the colon, allowing for the translocation of TNBS. This haptenization agent then interacts with amine groups of colon and/or microbiota proteins, binding trinitrophenyl groups to such proteins indiscriminately. Next, haptenized proteins are engulfed, processed and presented by APCs to T lymphocytes in the LP. This process, in the absence of appropriate regulatory mechanisms, leads to excessive secretion of IL-12 by mϕ and induction of an exaggerated Th1 response that, in turn, induces the production of other proinflammatory cytokines—such as TNF-α and IL-6—by mϕ, which are the immediate causes of inflammation (Fig. 5a) [34, 58].

Thus, it is possible that the intragastric administration of *L. lactis* MG1363 FnBPA^+^ (pValac::dts::*IL*-*4*) leads to entry of this bacterium into host cells, such as resident mϕ. This LAB is then lysed, and the pValac::dts::*IL*-*4* plasmid is released in the cytoplasm and translocated to the nucleus, where expression of the *IL*-*4* ORF occurs [59]. Next, secreted IL-4 contributes to the differentiation of Th2 cells, which also produce IL-4, thus cooperating with the restoration of the levels of this cytokine, which exerts a markedly inhibitory effect on the expression and release of Th1 cytokines [13]. Additionally, the presence of sufficient levels of IL-4 and bacterial products (either from invading *L. lactis* or intestinal microbiota) can programme the M2/hybrid mϕ phenotype in native M0 mϕ or can reprogramme the M2/hybrid mϕ phenotype in resident and/or M1 mϕ that accumulate during inflammation (Fig. 5b) [57, 60].

Differentiated M2/hybrid mϕ, as a group of highly specialized cells with characteristic gene expression, participate in tissue remodelling and repair and assist in the regulation of inflammation [60, 61]. This last function is probably related to M2/hybrid mϕ presenting an IL-10^hi^ phenotype, with these cells likely being responsible for the reestablishment of IL-10 levels observed in this study. Furthermore, IL-10-IL-10R interaction is crucial for the conditioning of mϕ in the intestinal mucosa because, in mice, the hyporesponsiveness of these cells to proinflammatory stimuli gradually develops during the maturation of monocytes, which, in turn, is correlated with the increase of IL-10 production. Thus, IL-10 could be considered one of the most important factors for mϕ quiescence to environmental stimuli. In addition, these mϕ seem to facilitate secondary expansion and maintenance of antigen-specific Foxp3^+^ Treg cells through IL-10 production [55] and consequently, to regulate TNBS-induced intestinal inflammation (Fig. 5b).

## Conclusions

Through the assessment of the therapeutic potential of *L. lactis* strains in a TNBS-induced murine model of colitis, it was observed that the invasive strain carrying the IL-4-coding vector was the only one that appeared to have played an immunomodulatory role in response to inflammation resulting from an exacerbated Th1 response. This outcome seemed to occur because *L. lactis* MG1363 FnBPA^+^ (pValac::dts::*IL*-*4*) was also the only strain that showed a tendency towards the restoration of IL-4 levels and that was capable of restoring IL-10 levels. The latter, in turn, was most likely due to the higher percentage of IL-10-producing regulatory cells (F4/80^+^MHCII^+^Ly6C^−^IL-10^+^, F4/80^+^MHCII^+^Ly6C^−^CD206^+^CD124^+^IL-10^+^, and CD4^+^Foxp3^+^IL-10^+^ cells) shown by the animals administered this strain.

Therefore, the invasive *L. lactis* strain carrying the IL-4-coding vector appeared to be the only one able to deliver the therapeutic plasmid in satisfactory quantities so that the *IL*-*4* ORF was transcribed, translated and secreted by host cells in levels sufficient to alter the intestinal environment. This outcome supported the idea that oral administration of *L. lactis* MG1363 FnBPA^+^ (pValac::dts::*IL*-*4*) has therapeutic potential and could be considered a promising alternative strategy for maintaining the anti-inflammatory and proinflammatory balance in the GI tract, especially in individuals affected by CD.

## Abbreviations

Ad5: human type 5 adenovirus
APC: allophycocyanin
APC: antigen-presenting cell
BGH polyA: polyadenylation signal sequence of the bovine growth hormone
BLG: bovine β-lactoglobulin
CD: Crohn’s disease
CFU: colony forming unit
CHO: Chinese hamster ovary
DAPI: 4′-6-diamidino-2-phenylindole
DSS: dextran sulphate sodium
DTS: DNA nuclear targeting sequence
FCS: foetal calf serum
FnBPA: fibronectin-binding protein A
Foxp3: forkhead box P3
GFP: green fluorescent protein
GI: gastrointestinal
GM17: M17 medium supplemented with 0.5 % glucose
HE: haematoxylin and eosin
HEPES: 4-(2-hydroxyethyl)piperazine-1-ethanesulfonic acid
HRP: horseradish peroxidase
IBD: inflammatory bowel disease
ICAM-1: intercellular adhesion molecule 1
IgE: immunoglobulin E
IFN-γ: interferon γ
IL-4: interleukin 4
IL-6: interleukin 6
IL-6R: interleukin 6 receptor
IL-10: interleukin 10
IL-12: interleukin 12
IL-17A: interleukin 17A
IMDM: Iscove’s Modified Dulbecco’s Medium
MCS: multiple cloning site
NF-ΚB: nuclear factor ΚB
NO: nitric oxide
LB: Luria–Bertani
LP: lamina propria
mϕ: macrophage
MPO: myeloperoxidase
mRNA: messenger RNA
NAG: *N*-acetyl-β-d-glucosaminidase
ORF: open reading frame
PBS: phosphate-buffered saline
pCMV: cytomegalovirus promoter
PCR: polymerase chain reaction
RELMα: resistin-like molecule α
RORγt: RAR-related orphan receptor γt
sIgA: secretory immunoglobulin A
SV40: Simian virus 40
T-bet: T-box expressed in T cells
TGF-β: transforming growth factor β
Th: T helper cell
TMB: 3,3′,5,5′-tetramethylbenzidine
TNBS: 2,4,6-trinitrobenzenesulfonic acid
TNF-α: tumour necrosis factor α
Treg: regulatory T cell
UC: ulcerative colitis
VCAM-1: vascular cell adhesion molecule 1
